# Hierarchical multi-view aggregation network for sensor-based human activity recognition

**DOI:** 10.1371/journal.pone.0221390

**Published:** 2019-09-12

**Authors:** Xiheng Zhang, Yongkang Wong, Mohan S. Kankanhalli, Weidong Geng

**Affiliations:** 1 State Key Laboratory of CAD&CG, College of Computer Science and Technology, Zhejiang University, Hangzhou, Zhejiang Province, China; 2 School of Computing, National University of Singapore, Singapore, Singapore; Newcastle University, UNITED KINGDOM

## Abstract

Sensor-based human activity recognition aims at detecting various physical activities performed by people with ubiquitous sensors. Different from existing deep learning-based method which mainly extracting black-box features from the raw sensor data, we propose a hierarchical multi-view aggregation network based on multi-view feature spaces. Specifically, we first construct various views of feature spaces for each individual sensor in terms of white-box features and black-box features. Then our model learns a unified representation for multi-view features by aggregating views in a hierarchical context from the aspect of feature level, position level and modality level. We design three aggregation modules corresponding to each level aggregation respectively. Based on the idea of non-local operation and attention, our fusion method is able to capture the correlation between features and leverage the relationship across different sensor position and modality. We comprehensively evaluate our method on 12 human activity benchmark datasets and the resulting accuracy outperforms the state-of-the-art approaches.

## 1 Introduction

Human Activity Recognition (HAR) refers to the automatic detection of various physical activities performed by people in their daily lives [1]. It has been applied to practical scenarios such as smart environment [2], health care [3], and footstep detection [4]. Benefiting from ubiquitous computing and well-protected individual privacy, sensor-based HAR research has received increasing interests recently.

In the sensor-based HAR task, the raw data from various modalities is collected and then utilized to infer useful contextual information for classifying activities. And there are two challenges lies in processing raw data. The first one is how to construct discriminative feature spaces from the heterogeneous sensor data. Focusing on this challenge, early methods leveraged human domain knowledge to feature engineering for HAR, and these properly designed *white-box features* are extracted based on different types of methods [5–9]. Recently, deep learning models have been brought significant impacts to HAR. Neural networks such as convolutional neural networks (CNNs) and recurrent neural networks (RNNs) are applied to learn *black-box features* from raw data [10–13]. We argue that these different types of features describe the contextual information of raw data from different viewpoints, and could be effectively integrated in a unified framework to take advantage of each other. The second challenge is how to integrate features from different feature spaces effectively, which can lead to more accurate and robust performance. We notice that the sensors are not only of different modalities, but also of different wearing positions on the human body. The heterogeneity in sensor modality and position inspired us to integrate views of feature spaces by a hierarchical context, i.e. from the aspect of feature level, position level and modality level.

Therefore, in this paper, we propose a hierarchical multi-view aggregation network for HAR, which can effectively fuse white-box features with black-box features from different feature spaces. Moreover, we design three aggregation modules to construct a unified representation for multi-view features from the aspect of feature level, position level and modality level. In feature level aggregation, we apply a non-local operation augmented with the *L*2-norm to explores the correlation between different features and fuse them. In the position level aggregation, we take the correlation of different sensor positions into consideration by introducing the correlation feature, which can enhance the representation of each view and effectively improves the resulting accuracy. Finally, in the modality level aggregation, we conduct a soft attention mechanism to quantify the discrimination of each view and fuse them for classification.

The main contribution of this paper is two-fold:

- We propose a Hierarchical Multi-View Aggregation Network (HMVAN) for HAR task, which targets at integrating features from various feature spaces. Specifically, our method constructs multi-view features for individual sensor from the point of view of white-box features and black-box features. These views are then embedded into a shared feature space and aggregated into a unified hierarchical representation. Compared with existing deep learning-based methods which mainly extracting black-box features from the raw data of different sensors, our model is much more effective in representing the discriminative information of human activities.
- We design three aggregation modules to integrate these views into a unified representation of multi-view features from the aspect of feature level, position level and modality level. Based on the idea of non-local operation and attention, our method is capable of capturing the correlation between features, and leveraging the relationship across different sensor position and modality, which effectively improves to the resulting recognition accuracy.

## 2 Related works

Sensor-based HAR task is usually formulated as a time-series segment classification problem. Segmentation by sliding windows, extraction of features followed by a classification constitute the standard pipeline for recognizing human activities. Previous works can be roughly divided into two categories: traditional feature-based methods and deep learning-based method. Conventional methods tend to using engineered features obtained by statistical process. Plötz et al. [5] calculated mean, standard deviation, energy and entropy for each source channel of accelerometers. And in [14], the authors extracted zero crossing, root mean square value, spectral energy, mean, variance, standard deviation, median and the sum of FFT (Fast Fourier Transformation) coefficients from accelerometers, gyroscopes and magnetometers. Chen and Shen [15] chose the feature set that includes mean, standard deviation, max, min, interquartile range, dynamic time warping distance, FFT coefficients and wavelet energy. Kwon et al. [16] explored how temporal structure can be add into distribution-based feature extraction schemes for improving performance of HAR applications. Although extracting and identifying relevant features is time-consuming, these approaches work relatively well, even when the data is scare and highly unbalanced [17].

Recently, deep learning methods are widely adopted for sensor-based HAR task for the capacity of extracting features without human domain knowledge. Convolutional neural network (CNN) has been widely used in many HAR works. The works such as [18] and [10], in which each axis of the sensor was treated as an independent channel and CNN was performed on every channel separately. But with 1D kernel, CNN can only capture local dependency over time. Therefore, in [19], different sensors were grouped by their position and 2D CNN was raised to capture both local dependency over time and spatial dependency over the sensors. Later in their work [20], they improved the networks by employing weight-sharing mechanism, which enabled the networks to learn modality-specific characteristics across multi-model sensor data. Jiang and Yin [21] assembled signal sequence of accelerometer and gyroscopes into a novel “activity image”, which enabled CNN to learn optimal features from the “activity image” for image classification task. In [22], pressure sensor data was converted into pressure distribution images, then CNN was used for transfer learning on the converted imagery data. Ravi et al. [23] introduced a deep learning method which combines convolutional features learned from inertial sensor data together with complementary information from a set of shallow features to enable accurate activity classification. Rueda et al. [24] presented three convolutional architecture to search for attributes that represent favorable signal segments for recognizing human activities.

In addition, recurrent neural network (RNN) shows competitive results when applied to HAR task. In [2, 25, 26], LSTM (Long Short-Term Memory) cell is the most used in RNN-based architecture. Edel et al. [27] proposed a binarized-BLSTM-RNN model, of which the input, weight parameters and intermediate hidden layer output were all binary-valued. Vu et al. [28] developed a self-gated recurrent neural network, which reduced resource memory usage and computational cost. In [29], different convolutional and recurrent models were evaluated and bi-directional LSTMs outperformed the others. To better interpret the recurrent networks to gain insight into the models’ behavior, Zeng et al. [30] introduced temporal attention and sensor attention into RNN, adaptively focusing on important signals and sensor modalities.

Moreover, there are some hybrid deep architectures which combines different models. An Early work is [31], which recommended a deep neural network for modeling the emission distribution of hidden Markov models. A good example that combines CNN and RNN was offered by [11]. It was proven that the performance of the proposed deepConvLSTM was better than single CNN. Yao et al. [32] proposed DeepSense, which integrates convolutional and recurrent neural networks to exploit local interactions of different sensory modalities. Zheng et al. [33] combined CNN with stacked auto-encoder while Liu et al. [34] combined CNN with restricted Boltzmann machine.

The method proposed in [23] is most related to ours, where they combined a set of shallow features with those obtained from deep learning. Our method has two key differences. Firstly, we construct different views of feature spaces on multi-modal sensor data rather than simply directly extracting single view features. The “multi-view” fashion can better represent the specific property of each type of sensor data. Secondly, our method introduces three aggregation modules to integrate these views into a unified representation of multi-view features from the aspect of feature level, position level and modality level. Comparing with simply concatenating different features, our approach is capable of capturing the correlation between features, and leveraging the relationship across different sensor position and modality, which effectively improves to the resulting recognition accuracy.

## 3 Problem formulation

Sensor-based HAR task is usually formulated as a time-series segment classification problem. Given a HAR dataset *S*, we denote the samples generated by sliding window procedure as $I={\left\{\left(A_{i}^{s},G_{i}^{s},M_{i}^{s}\right)\right\}}_{i=1}^{N_{s}}$, in which $A_{i}^{s}$, $G_{i}^{s}$ and $M_{i}^{s}$ represent the data of accelerometers, gyroscope and magnetometer respectively. In addition, $A_{i}^{s}\in R^{P\times 3}$, $G_{i}^{s}\in R^{P\times 3}$, and $M_{i}^{s}\in R^{P\times 3}$ where *P* is the amount of positions of accelerometers, gyroscopes and magnetometers. Each sensor has 3 channels. The number of modalities *V* = 3.

For each sample *I*, we aim to map the raw data into a unified representation for multi-view features **h** by the following function:

$$\begin{array}{c} h=G_{m}(G_{p}\left(G_{f}\left(C\left(A^{s},G^{s},M^{s}\right)\right)\right) \end{array}$$ (1)

where *C*(⋅) is the construction of multiple views. *G*_*f*_, *G*_*p*_ and *G*_*m*_ is the feature level, position level and modality level aggregation, respectively.

Given the unified representation **h**, we simultaneously optimize the network by minimizing a loss function *L* to shorten the distance between the predicted label and ground truth. In the following sections, we will present details of the construction of multi-view features, the design and optimization of the aggregation module of each layer.

## 4 Hierarchical multi-view aggregation network

**Fig 1:**
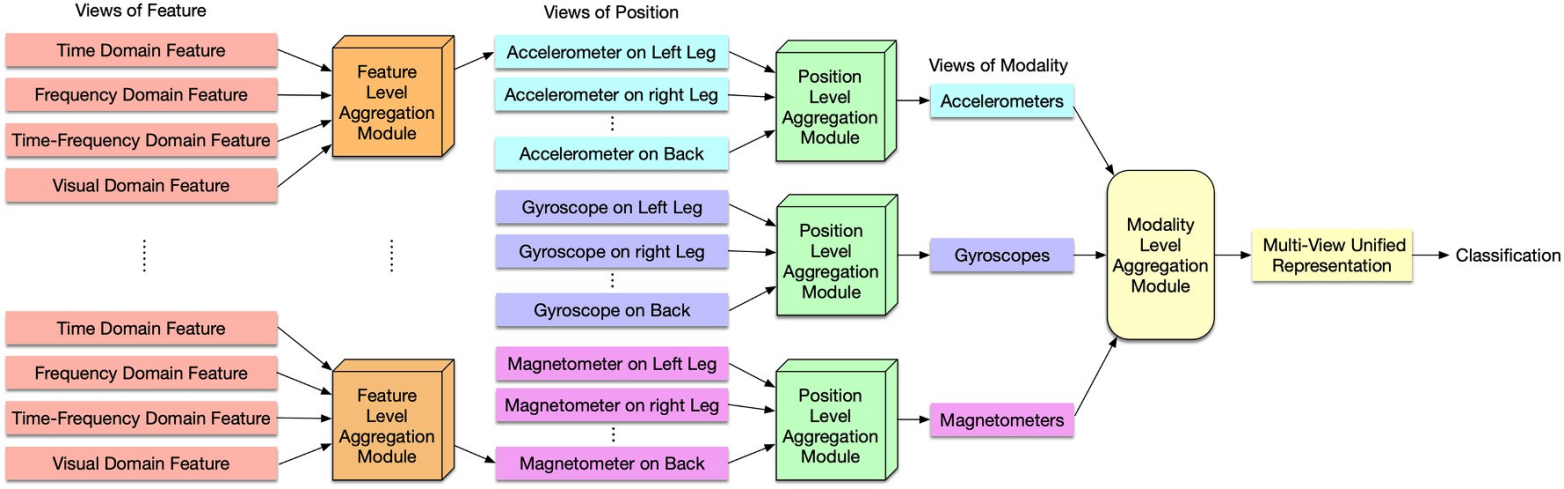
An overview of the hierarchical multi-view aggregation network. We first construct four views of feature spaces for each individual sensor in the bottom layer. Then we designed three aggregation modules to integrate views step-by-step into a multi-view unified representation.

Our proposed hierarchical multi-view aggregation network is a three-layer multi-view framework, as illustrated in Fig 1. First of all, on the bottom layer, four views of features, including time domain, frequency domain, time-frequency and visual domain features, are extracted to characterize the measurement of a sensor in a certain position. Secondly, views of features are aggregated by feature level aggregation to form the views of position in the middle layer. Then position level module aggregates the views of positions into a view of modality, and the unified representation of multi-view features is obtained by aggregating views of modality. Finally, cross-entropy losses for each view of modality and the unified representation are computed to optimize the network. The detail of each component is explained in the following subsections.

### 4.1 Multi-view construction

The white-box features extracted for HAR task is obtained by the statistical process, and can be divided into three main categories namely as time domain features, frequency domain features and time-frequency domain features [8]. Time domain features are extracted from the time series of the raw data, while frequency domain features are obtained from the frequency representation of the data. And when a wavelet transformation is applied to the raw data, the extracted features are in the time-frequency domain. These features from different feature spaces represent the statistical property of the data’s time domain, frequency domain and time-frequency domain. Recently, [21] and [22] proposed to transform non-interpretable sensor data into the image domain and applied deep neural networks to extract features. Specifically, the segments of sensor data are transformed into a visually interpretable image and CNN was trained to discriminate the images of different activities. Therefore, we add a view of visual domain features as the black-box feature, complementing to the white-box features.

In our method, we use three views of white-box features and one view of black-box feature to from the views of features. We summarize the white-box features [5, 8, 14, 15] in Table 1. In our case, we calculate resultant acceleration and angular velocity from the data of accelerometer and gyroscope respectively and treat them as channels. And we apply FFT and wavelet transformation to transform the time-domain data into the frequency domain and the time-frequency domain. For visual domain feature, we follow the method in [21]. The segment of sensor data is transformed into an activity image, and CNNs is trained to extract black-box features from the images. Specifically, raw signals are first stacked row by row into a signal image by an algorithm. In the signal image, every signal sequence has the chance to be adjacent to every other sequence, which enables CNN to extract hidden correlation between neighboring signals. Then 2D discrete Fourier transformation is applied to the signal image and its magnitude is chosen as activity image. The output dimension of CNN is 120. After multi-view construction, we obtain a multi-view representation ${\left\{{\left\{x^{\text{time}},x^{\text{fre}},x^{\text{t-f}},x^{\text{visual}}\right\}}_{p=1}^{P}\right\}}_{v=1}^{V}$ of the raw data.

**Table 1:** White-box features extracted from the sensor data.

Domain	Features
Time	Interquartile Range, Max, Min, Mean, Median
	Amplitude, Mean Crossing, Signal Magnitude Area
	Standard Deviation, Skewness, Kurtosis, Zero Crossing
Frequency	Largest Frequency Component, Energy
	Skewness, Kurtosis, Weighted average
	Sum of the First 5 FFT Coefficients
Time-frequency	Standard Deviation, Max, Min, Mean
	Median Crossing Rate, Wavelet Energy

### 4.2 Feature level aggregation

**Fig 2:**
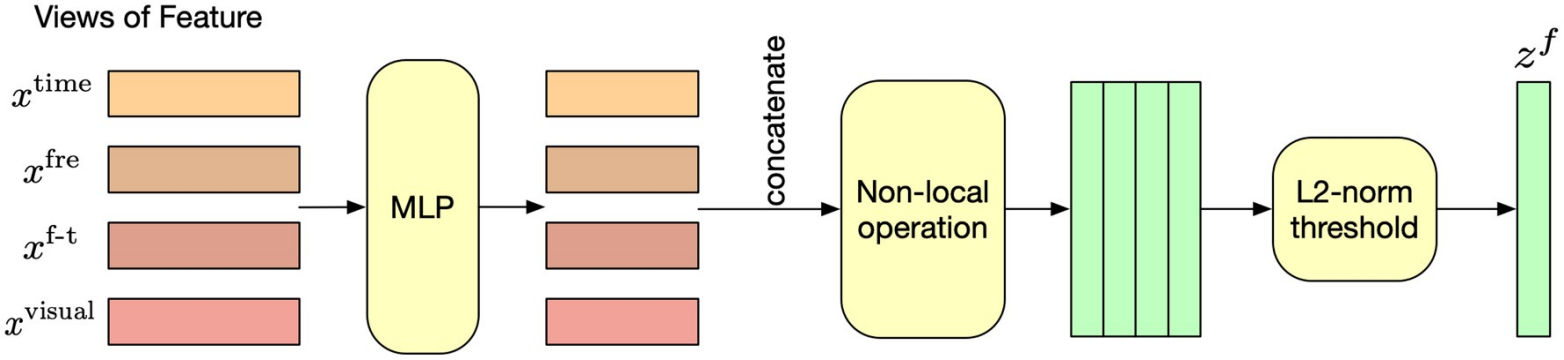
Overview of feature level aggregation.

In this subsection, a fusion module is proposed to aggregate the views of features, as shown in Fig 2. Given the *x*^time^, *x*^fre^, *x*^t-f^ and *x*^visual^ from a single sensor in a certain position. We first embed *x*^time^, *x*^fre^, *x*^t-f^ and *x*^visual^ into a shared feature space, i.e.,

$$\begin{array}{cc} {\hat{x}}^{\text{time}} & ≔\text{MLP}\left(x^{\text{time}};\theta^{\text{time}}\right)\in R^{d} \end{array}$$ (2)

$$\begin{array}{cc} {\hat{x}}^{\text{fre}} & ≔\text{MLP}\left(x^{\text{fre}};\theta^{\text{fre}}\right)\in R^{d} \end{array}$$ (3)

$$\begin{array}{cc} {\hat{x}}^{\text{t-f}} & ≔\text{MLP}\left(x^{\text{t-f}};\theta^{\text{t-f}}\right)\in R^{d} \end{array}$$ (4)

$$\begin{array}{cc} {\hat{x}}^{\text{visual}} & ≔\text{MLP}\left(x^{\text{visual}};\theta^{\text{visual}}\right)\in R^{d} \end{array}$$ (5)

where MLP stands for a multilayer perceptron and *d* is the dimensionality of the shared feature spaces.

Inspired by non-local neural networks [35], we then concatenate all those features into a matrix $x\in R^{d\times 4}$ and transform it into a fused multi-feature embedding *z* by non-local operation:

$$\begin{array}{c} \hat{x}=\text{softmax}\left(x^{T}W_{1}^{T}W_{2}x\right)W_{g}x \end{array}$$ (6)

$$\begin{array}{c} z=W_{z}\hat{x}+x \end{array}$$ (7)

where $W_{1}^{T}$ and *W*_2_ are learnable parameters for linear transformation.

Since the non-local operation does not change the dimension of the input vector, the dimension of the multi-feature embedding *z* could be relatively large and result in over-fitting. Motivated by [36], we adopt *L*2-norm to select subsets of *z* for avoiding over-fitting. Firstly, a vector $p\in R^{4}$ was computed to measure the importance of each row in *z*:

$$\begin{array}{c} p_{i}≔∥z_{i}{∥}_{2} \end{array}$$ (8)

where *z*_*i*_ means the *i*-th row in *z*. Then we select the rows in *z* through a threshold. The key idea is to make the vector *p* (the norm of the embedding at each location) “compete” against a threshold *τ*. We put both part into the competition by selecting those elements of *z* whose softmax of *p* values exceed the threshold, i.e.:

$$\begin{array}{c} z^{f}=\left[z_{l_{1}},...,z_{l_{k}}\right]\in R^{k},\;\text{where}\;l_{i}:\text{softmax}\left(p_{i}\right)>\tau \end{array}$$ (9)

Although *τ* could be chosen through the hyper-parameter selection, we follow the determination of the threshold *τ* ≔ 1/*d* in [36]. Such value for *τ* has an interesting interpretation. If each location of the input were equally important, we would sample the locations from a uniform probability distribution *p*(⋅) ≔ *τ* = 1/*d*. This is equal to a probability distribution induced by the vector *p* of a neural network with uniformly distributed representation, i.e. *τ* = softmax(*p*_uniform_), and hence the trained network with the vector *p* has to “win” against the *p*_uniform_ of the random network in order to select right input features by shifting the probability mass accordingly [36].

Finally, *z*^*f*^ is the aggregated feature obtained from feature level aggregation module, and it is further fed into position level aggregation module.

### 4.3 Position level aggregation

In this subsection, we introduce the position level aggregation module to fuse the features from the feature level aggregation module according to the position of sensors. Nowadays, the exploration of correlation has been proven to be successful in achieving good performance in computer vision tasks such as visual question answering [37] and object detection [38]. We believe that the correlations between the sensors in different position can also help improve performance. Intuitively, in a certain type activity, the correlation between some sensors is bigger than the others. For example, when people walk, the state of the accelerometer on the left leg is similar to the state of the accelerometer on the right leg, but it is different from the state of the accelerometer on the back [39]. Therefore, we take the correlation between the sensors into the position level aggregation process.

**Fig 3:**
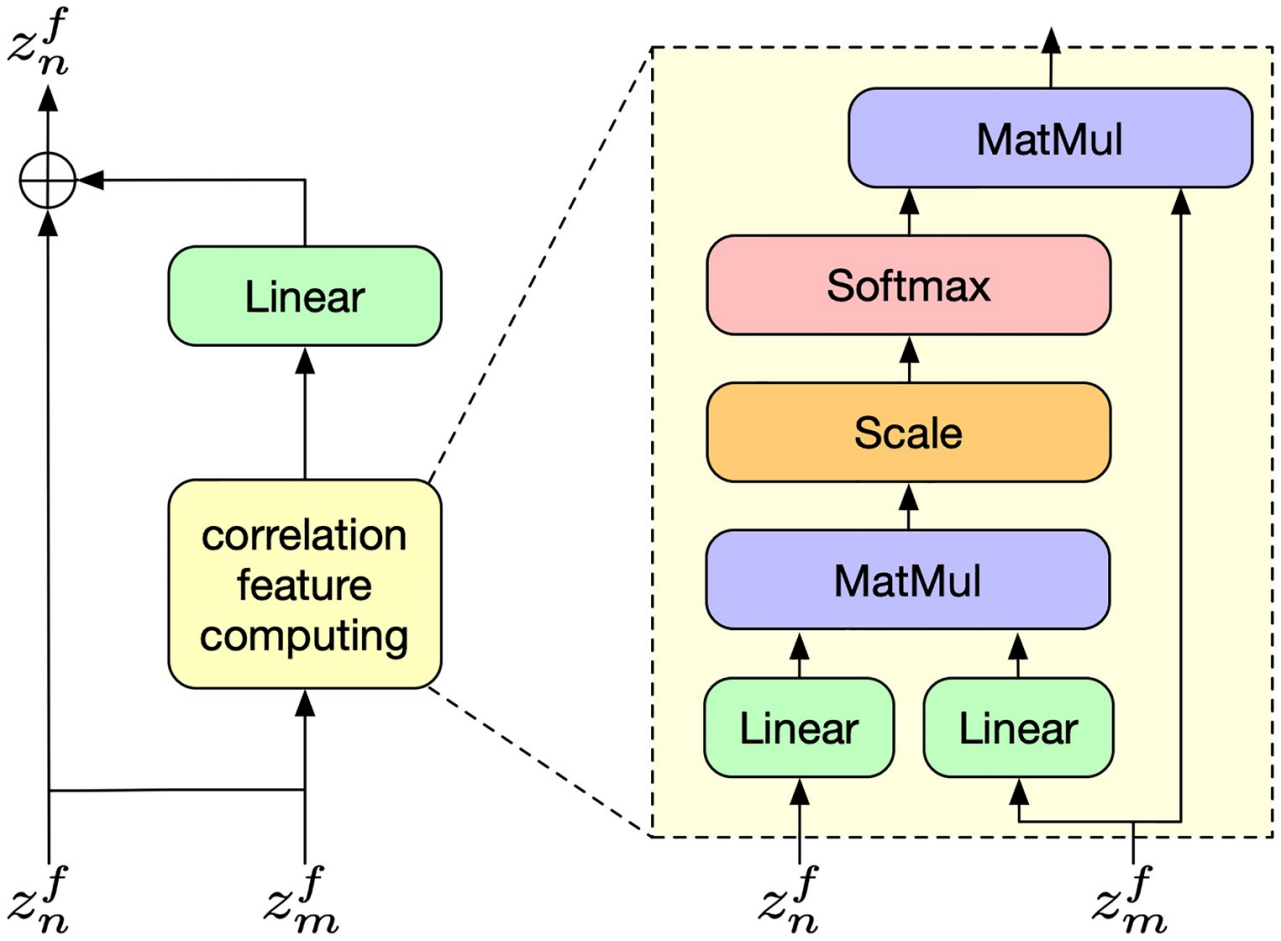
Overview of correlation feature computing.

Inspired by [38], we define a correlation feature *f*_*cor*_(*n*) for the *n*-th view of position, as shown in Fig 3, which is computed by the following equation:

$$\begin{array}{c} f_{cor}\left(z_{n}^{f}\right)=\sum_{m}\alpha^{mn}\cdot \left(W_{V}\cdot z_{m}^{f}\right) \end{array}$$ (10)

In fact, the correlation feature for the *n*-th view is a weighted sum of the features from the other views. The weights *α*^*mn*^ measures the correlation between the *n*-th view and the *m*-th view, which can be calculated as follows:

$$\begin{array}{c} \alpha^{mn}=\text{softmax}(\frac{{\left(z_{m}^{f}\right)}^{T}W_{m}^{T}W_{n}z_{n}^{f}}{\sqrt{d}}) \end{array}$$ (11)

where *d* is the dimension of features of each view. When the value of *d* is relatively big, the dot products grow large in magnitude, pushing the softmax function into regions where it has extremely small gradients [40]. To counteract this effect, we scale the dot product by $1/\sqrt{d}$.

Then we add the correlation feature to the view’s original features. For the *n*-th view, that is:

$$\begin{array}{c} z_{n}^{f}=z_{n}^{f}+f_{cor}\left(n\right) \end{array}$$ (12)

As a result, each view was enhanced by the correlation feature, which is an aggregation of the other views by the correlation between them. At last, the result of position level aggregation *z*^*p*^ is obtained by a view pooling operation, i.e. element-wise max-pooling, to all the views.

### 4.4 Modality level aggregation

Given the features after position level aggregation, the objective here is to conduct a modality level aggregation towards a final representation for classification. We apply a soft attention mechanism to compute an aggregated vector *h* over the input *z*^*p*^:

$$\begin{array}{c} h=\sum_{v}\beta_{v}z_{v}^{p} \end{array}$$ (13)

The weight *β*_*v*_ for each modality is computed by:

$$\begin{array}{cc} u_{v} & =\text{tanh}(W_{u}z_{v}^{p}+b_{u}) \end{array}$$ (14)

$$\begin{array}{cc} \beta_{v} & =\frac{\text{exp}(u_{v})}{\sum_{v}\text{exp}\left(u_{v}\right)} \end{array}$$ (15)

In this way, the view containing more discriminative information contribute more to the final representation.

By using the three levels of aggregation in our hierarchical multi-view framework, a unified representation *h* for multi-view features can be constructed, and the correlation between features and position of sensors is leveraged to help get a more effective representation of the discriminative information of human activities.

### 4.5 Loss function

Given the final representation *h* of a sample *I*, we can compute the probability of a sample belonging to each class:

$$\begin{array}{c} p_{i}=\text{softmax}\left(W_{h}h_{i}+b_{h}\right) \end{array}$$ (16)

And we use cross-entropy to calculate the classification loss:

$$\begin{array}{c} L_{\text{final}}=-\sum_{i}y_{i}log\left(p_{i}\right) \end{array}$$ (17)

To further strengthen the representation capability of our model, we add auxiliary losses to the network. For each view of modality, we calculate a cross entropy loss:

$$\begin{array}{cc} p_{v,i} & =\text{softmax}(W_{p}z_{v,i}^{p}+b_{p}) \end{array}$$ (18)

$$\begin{array}{cc} L_{\text{auxiliary}} & =-\sum_{i}y_{i}log\left(p_{v,i}\right) \end{array}$$ (19)

where *y*_*i*_ represents the ground truth of sample *I*. Therefore, the total loss L of our model is the weighted sum of $L_{\text{final}}$ and $L_{\text{auxiliary}}$ for each view of modality:

$$\begin{array}{cc} L & =L_{\text{final}}+L_{\text{auxiliary}} \end{array}$$ (20)

$$\begin{array}{cc} & =-(\sum_{i=1}^{N_{s}}\sum_{v=1}^{V}y_{i}\text{log}\left(p_{v,i}\right)+\sum_{i=1}^{N_{s}}y_{i}\text{log}\left(p_{i}\right)) \end{array}$$ (21)

## 5 Experiments

### 5.1 Benchmark datasets and experiment setup

To demonstrate the adaptability of our method, we conduct a number of experiments on 12 public human activity datasets. The details about the datasets are shown in Table 2. For these datasets, we divide them into four categories:

1. Datasets which contain multiple modalities and positions, including OPPORTUNITY, PAMAP2, DSA, MHEALTH and HHAR.
2. Datasets which contain multiple positions, but only one single modality, including Skoda and Daphnet Gait.
3. Datasets which contain multiple modalities, but only one single position, including UCI Smartphone, USC-HAD, SHO.
4. Datasets which contain only one single modality and one single position, including WISDM v1.1 and WISDM v2.0.

**Table 2:** Public human activity datasets for evaluation.

Dataset	#Subject	Sample Rate	#Activity	#Sample	Sensor	#Position	Reference
OPPORTUNITY	4	32Hz	16	191564	A, G, M	5	[41]
PAMAP2	9	100Hz	18	64173	A, G, M	3	[42]
DSA	8	25Hz	19	75998	A, G, M	5	[43]
MHEALTH	10	50Hz	12	40522	A, G, M	3	[3]
HHAR	9	100-200Hz	6	366038	A, G	3	[39]
Skoda	1	96Hz	10	22000	A	4	[44]
Daphnet Gait	10	64Hz	2	49942	A	3	[45]
UCI Smartphone	30	50Hz	6	10299	A, G	1	[46]
USC-HAD	14	100Hz	12	41998	A, G	1	[47]
SHO	10	50Hz	7	20998	A, G, M	1	[14]
WISDM v1.1	29	20Hz	6	91515	A	1	[48]
WISDM v2.0	36	20Hz	6	248653	A	1	[49]

A = accelerometer, G = gyroscope, M = magnetometer

For the first type of datasets, we can apply the proposed method without any changes. For the second type, since there is only one sensor modality, we can only construct a two-layer multi-view model without views of modality. Similarly, we construct a two-layer multi-view model without views of position. And for the last type, there is only one single modality and one single position. Therefore, our method is simplified to a basic one-layer multi-view model. On the OPPORTUNITY dataset, we use the same training set, validation set and test set employed in the OPPORTUNITY challenge to train and test our models, as all the other papers did. On the other dataset, we conduct a 5-fold cross validation to study the performance of our model, since there is no agreed division on the datasets.

### 5.2 Implementation details

Sensor data are preprocessed to fill in missing values using linear interpolation and to do a per channel normalization to interval [0, 1]. The length of sliding window is 1.2 second and the overlap is 50%. We initialize the CNN for extracting visual features after training on ImageNet, and finetune it on respective dataset, following the architecture of CNN in [21]. The aggregation networks are trained on each dataset, and finetuned along with the CNN. The output size of multilayer perceptron is 128, and dropout is applied to the output with probability of 0.2. The weight decay of the whole network is configured to 0.0001. During the training, we apply a mini-batch size of 128 samples and an Adam optimizer [50] with a learning rate of 0.001 to train the networks. These hyper-parameters are same for all HAR datasets. The whole pipeline is implemented using TensorFlow [51].

### 5.3 Evaluation metrics

We adopt the accuracy and the weight *F*_1_ score as our evaluation metrics for fair comparison with the state-of-the-arts. Since the classes of the datasets may be highly unbalanced, the weight *F*_1_ score can also measure the performance of the model appropriately. Specifically, the weight *F*_1_ score is defined as follows:

$$\begin{array}{c} F_{1}=\sum_{i}2*w_{i}\frac{{\text{precision}}_{i}\cdot {\text{recall}}_{i}}{{\text{precision}}_{i}+{\text{recall}}_{i}} \end{array}$$ (22)

where *i* is the class index and *w*_*i*_ = *n*_*i*_/*N* is the proportion of samples of class *i*, with *n*_*i*_ being the number of samples of the *i*-th class and *N* being the total number of samples.

### 5.4 Overall performance

In previous methods, benchmark datasets used for evaluation are quite different. In order to make a fair comparison, we implement five state-of-the-art methods and conduct comprehensive experiments on 12 datasets. In experiments, our HMVAN is compared to [21], [11], [29], [23] and [24]. In addition, we provided the results of the simplified version of our HMVAN on the Datasets which contain multiple modalities or positions. The overall comparison results are presented in Table 3. It is illustrated that our method is capable of achieving higher performance on these datasets. First of all, on the datasets which have multiple modalities and positions, such as the OPPORTUNITY dataset, our method improves the *F*_1_ score from 0.929 to 0.933 for gesture recognition task, and from 0.900 to 0.917 for locomotion recognition task, when compared with [24]. And on the second type of datasets which only have one single modality, such as Skoda dataset, our method improves the state-of-the-art [11] from 0.958 to 0.965. Then on the third type of datasets which only have one single position, such as UCI Smartphone dataset, our method improves the accuracy from 0.9518 to 0.955 when compared with [21]. On the last type of dataset, which only have one single modality and one single position, such as WISDM dataset v1.1, our method can still outperform the other methods, and brings 0.4% improvement to the result of [23]. Moverover, it is shown that on the dataset with multiple modalities or positions, our simplified HMVAN can still get competitive results with those state-of-the-art methods. Note that our method outperforms the method proposed in [23], which is most related to ours, which demonstrated that our model is benefited from integrating black-box features with white-box features in a hierarchical multi-view structure and get better performance.

**Table 3:** Comparison of the proposed model against the state-of-the-art methods on various human activity benchmark datasets.

	Results from each method													
	Jiang et al. [21]		DeepConvLSTM [11]		Hammerla et al. [29]		Ravi et al. [23]		attrCNN-IMU [24]		HMVAN		Simplified HMVAN	
Datasets	Acc.	*F*_1_	Acc.	*F*_1_	Acc.	*F*_1_	Acc.	*F*_1_	Acc.	*F*_1_	Acc.	*F*_1_	Acc.	*F*_1_
OPP-Gesture	0.913	0.912	-	0.915*	-	0.927*	0.922	0.921	-	0.929*	**0.934**	**0.933**	0.930	0.929
OPP-Locomotion	0.889	0.889	-	0.895*	0.892	0.891	0.897	0.896	-	0.900*	**0.918**	**0.917**	0.902	0.902
PAMAP2	0.911	0.910	0.927	0.926	-	0.937*	0.930	0.929	-	0.9088*	**0.944**	**0.943**	0.932	0.932
DSA	0.863	0.862	0.877	0.872	0.892	0.891	0.884	0.883	0.865	0.865	**0.905**	**0.904**	0.885	0.885
HHAR	0.954	0.954	0.977	0.976	0.959	0.958	0.940	0.940	0.947	0.947	**0.978**	**0.977**	0.953	0.952
MHEALTH	0.933	0.932	0.921	0.920	0.946	0.945	0.950	0.949	0.942	0.941	**0.968**	**0.967**	0.951	0.950
Skoda	0.944	0.943	-	0.958*	0.950	0.948	0.953	0.952	0.959	0.958	**0.966**	**0.965**	0.954	0.954
Daphnet Gait	0.901	0.899	0.942	0.941	-	0.760*	0.958*	-	0.933	0.932	**0.966**	**0.965**	0.960	0.959
UCI Smartphone	0.9518*	-	0.944	0.944	0.931	0.930	0.945	0.943	0.950	0.950	**0.955**	**0.954**	0.947	0.947
USC-HAD	0.9701*	-	0.957	0.957	0.954	0.953	0.961	0.959	0.967	0.965	**0.975**	**0.973**	0.964	0.963
SHO	0.9993*	-	0.987	0.986	0.989	0.989	0.994	0.994	0.997	0.997	**0.9995**	**0.9987**	0.9958	0.9958
WISDM v1.1	0.955	0.954	0.948	0.947	0.933	0.933	0.986*	-	0.966	0.965	-	-	**0.990**	**0.989**
WISDM v2.0	0.897	0.896	0.906	0.905	0.911	0.911	0.927*	-	0.920	0.919	-	-	**0.931**	**0.930**

Results marked with ‘*’ are obtained from the papers.

### 5.5 Ablation study

In this section, we conduct ablation experiments to study the effectiveness of individual component in our model on the validation dataset of the OPPORTUNITY dataset for locomotion recognition task.

#### 5.5.1 On the hierarchical multi-view structure

The primary contribution to be investigated is the effectiveness of the hierarchical structure multi-view structure for human activity recognition. We compare the HMVAN with three baselines, which removes the feature aggregation layer, position aggregation layer and modality aggregation layer respectively. Moreover, we also drop the $L_{\text{auxiliary}}$ of the HMVAN as a baseline to explore the improvement by the auxiliary losses for each view of modality. As shown in Table 4, the *F*_1_-score declines 1.6%, 3.3% and 2.5% when removing the feature aggregation layer, the position aggregation layer and the modality aggregation layer from HMVAN respectively. Such decrease in performance shows the hierarchical multi-view structure indeed plays an important role in our proposed method. In addition, adding auxiliary losses to HMVAN brings an improvement of 1.2%, which demonstrates its ability to strengthen the representation capability of our HMVAN. From the results, we can infer that, on the dataset which contain multiple modalities and positions, the model is benefited more from the position aggregation layers than feature aggregation layer. The result declines 3.3% when not using position aggregation layer, while the result declines 1.6% when not using feature aggregation layer, which is smaller than the former.

**Table 4:** Results of our model with different structure.

Methods	Accuracy
HMVAN w/o position aggregation layer	0.874
HMVAN w/o modality layer	0.882
HMVAN w/o feature aggregation layer	0.891
HMVAN w/o auxiliary losses	0.895
HMVAN	0.907

#### 5.5.2 On different views

We illustrate the effectiveness of each view of features in HMVAN. We exclude each view of features in each layer from HMVAN one by one, and the result is reported in Table 5. From the result, we can see that each view of features brings more or less improvement to HMVAN. The view of frequency domain features contributes most to our model, which brings 2.6% improvements. The view of time domain features and the view of time-frequency domain features contribute 2.2% and 1.7% improvements to the HMVAN respectively. In addition, if we exclude the view of visual domain features, the performance of our model decreases 2.1%. Therefore, it can be inferred that the view of visual domain features is effective to improve the performance of our model.

**Table 5:** Results of the proposed model with different views.

Methods	Accuracy
HMVAN w/o time domain features view	0.885
HMVAN w/o frequency domain features view	0.881
HMVAN w/o time-frequency domain features view	0.890
HMVAN w/o visual domain features view	0.886
HMVAN	0.907

#### 5.5.3 On the feature level aggregation

We further compared different methods in the feature level aggregation module. The non-local operations are replaced by four different operations. And we remove the *L*2-norm to prove its effectiveness of preventing over-fitting. As shown in Table 6, the non-local operation achieves better performance than the other feature aggregation methods, which proves that our model can gain benefit from the feature correlation learned from the non-local operation. In addition, the *L*2-norm plays an important role to regularize the network, since it brings 0.7% improvements to the model only using non-local operation.

**Table 6:** Results of different feature level aggregation methods.

Methods	Accuracy
element-wise adding	0.885
element-wise multiplying	0.881
element-wise mean	0.890
element-wise maximum	0.886
non-local	0.900
*L*2-norm	0.863
non-local + *L*2-norm	0.907

#### 5.5.4 On the position level aggregation

In position level aggregation, the correlation feature plays an important role. We drop the correlation feature to prove its contribution to our model. As is illustrated in Table 7, simple view pooling cannot effectively find the correlation between different position and only keeps a part of discriminative information. By adding correlation feature, each view is augmented with the correlation between position, which leads to a better performance in accuracy.

**Table 7:** Results of different position level aggregation methods.

Methods	Accuracy
simple view pooling	0.875
correlation feature + view pooling	0.907

#### 5.5.5 On the modality level aggregation

For modality level aggregation, we compared our method with five different approaches: MLP, element-wise adding, element-wise multiplying, element-wise mean and element-wise maximum. It can be seen in Table 8 that our attention aggregation method performs better than the other methods. It is able to fully explore the contribution of each modality to the final representation.

**Table 8:** Results of different modality level aggregation methods.

Methods	Accuracy
MLP	0.894
element-wise adding	0.899
element-wise multiplying	0.881
element-wise mean	0.887
element-wise maximum	0.901
attention fusion	0.907

#### 5.5.6 On the attention mechanisms

In the proposed framework, we use three types of attention mechanisms in different levels of aggregation. To explore the effectiveness of each type of attention mechanism in the particular layer, we conduct experiments to interchange them in each layer. We define the attention mechanism in feature, position and modality aggregation layer as attention type #1, type #2 and type #3 respectively. The results are presented in Table 9. It is shown that when we interchange the different attention mechanisms the results declined heavily. Since the goal of each layer is different, we properly design each aggregation layer to enhance the performance of the whole model. Firstly, on the feature aggregation layer, the model aims to learn the relationship between the different views of feature spaces. Since the dimension of each feature space is relatively large, we use the non-local operation to effectively compute the interact of features. And *L*2-norm regularization is added to the output for preventing over-fitting. Secondly, on the position aggregation layer, our goal is to learn a correlation features which represent the dependency of each position with the others. Then the correlation features are added to the original layer to enhance the features of each view, therefore each view actually contains holistic information of the whole position. At last, on the modality aggregation layer, our method intends to combine the features of each view of modality according to their contribution to the final representations, and simple soft attention is used to calculate the weight of each view. Although it is feasible to interchange these attention mechanisms in the programming, the performance of the model will decline when we use an improper attention mechanism.

**Table 9:** Results of different attention mechanisms in each aggregation layer.

Feature layer	Position layer	Modality layer	Accuracy
type #1	type #1	type #1	0.837
type #2	type #2	type #2	0.802
type #3	type #3	type #3	0.849
type #1	type #2	type #1	0.875
type #1	type #3	type #1	0.844
type #1	type #1	type #2	0.852
type #1	type #1	type #3	0.850
type #1	type #2	type #2	0.822
type #2	type #2	type #2	0.784
type #3	type #2	type #2	0.802
type #2	type #1	type #2	0.801
type #2	type #3	type #2	0.823
type #2	type #2	type #1	0.795
type #2	type #2	type #3	0.809
type #1	type #3	type #3	0.867
type #2	type #3	type #3	0.871
type #3	type #3	type #3	0.880
type #3	type #1	type #3	0.865
type #3	type #2	type #3	0.883
type #3	type #3	type #1	0.870
type #3	type #3	type #2	0.842
type #1	type #2	type #3	**0.907**
type #3	type #2	type #1	0.821
type #2	type #1	type #3	0.812

#### 5.5.7 On the number of training samples

**Fig 4:**
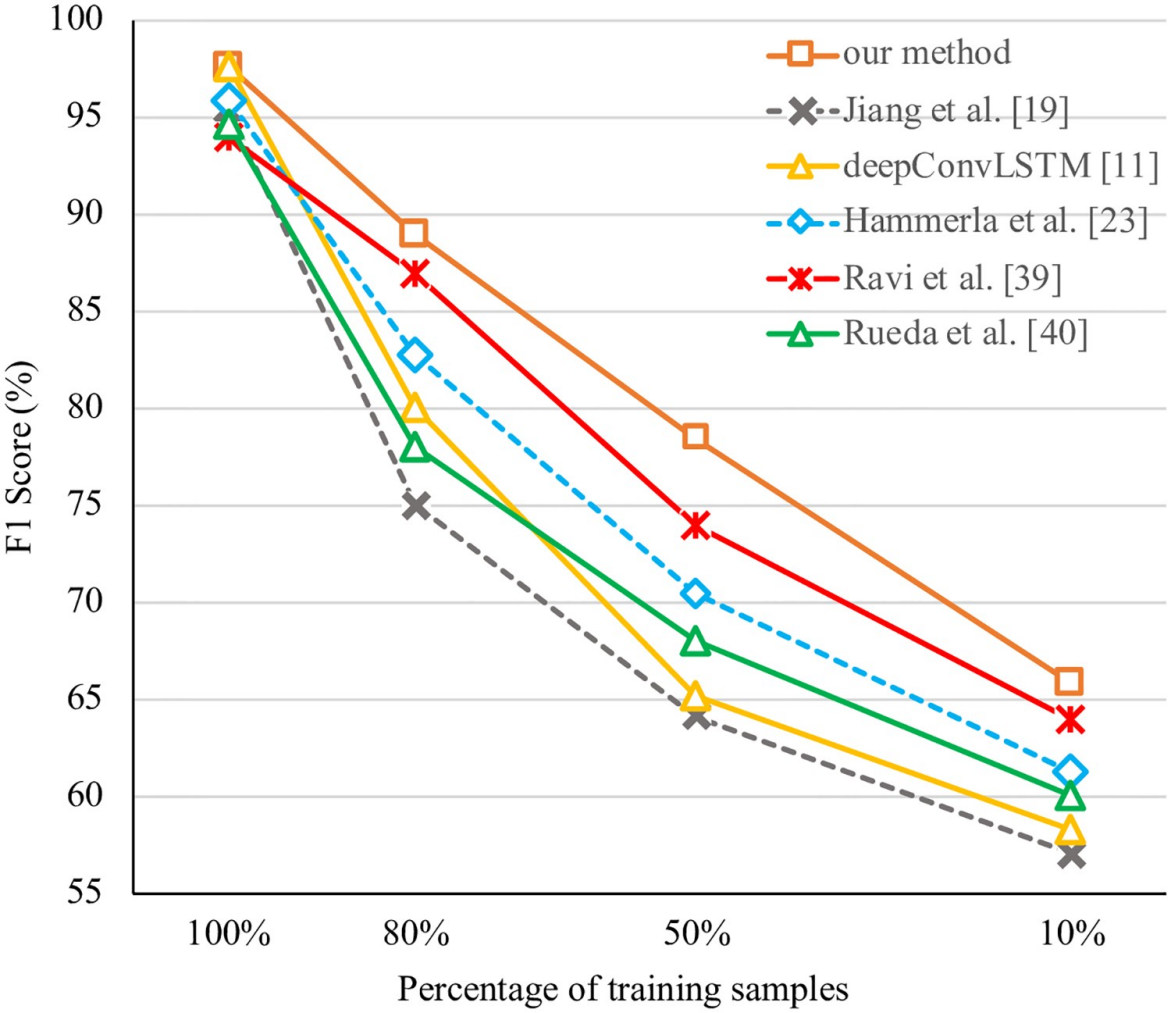
Comparison of the proposed model against the state-of-the-art methods with different percentage of training samples in HHAR dataset.

To further compare the performance of our method with the “pure” deep learning methods, we conduct experiments on HHAR dataset with different percentage of training samples to see how the performance changes. Specifically, we provide 10%, 50%, 80% and 100% of training samples to train each model and evaluate them on the test dataset. The comparison of our method with the other state-of-the-art methods are in Fig 4. It is shown that the performance our method still performs better than the other state-of-the-art methods when only using a small percentage of the training samples.

## 6 Discussion

The main findings from the direct comparison of our HMVAN against the other methods which treat sensor data as single view is that: (1) HMVAN reaches higher performance of both accuracy and *F*_1_ score; (2) it is significantly better able to disambiguate closely-related activities; (3) it is applicable even when the dataset is relatively small. Firstly, these findings support the hypothesis that white-box features and black-box features could be effectively integrated in a unified framework to take advantage of each other. Note that even when the dataset only contains one single modality and one single position, our method can still get a better performance than the other methods. The results demonstrate that the model is benefited from the construction of different views of feature spaces on multi-modal sensor data. Secondly, it has been proved that capturing the feature dependency is important to fine-grained action classification [11]. In our method, we introduce three attention mechanisms to capture the correlation between features, different sensor position and different modality, which effectively improves to the resulting recognition accuracy. At last, previous study have shown that shallow features can perform relatively well when the dataset is scale and unbalanced [17]. The results of our model with different percentage of training samples shows that our method outperforms those “pure” deep learning method when the dataset is insufficient, which demonstrate different views of shallow feature indeed help our model to gain competitive results on small dataset.

The confusion matrices on the OPPORTUNITY dataset for the gesture recognition task are illustrated in Table 10 for our HMVAN and Table 11 for the method in [23]. The confusion matrices contain information about actual and predicted gesture classifications, to identify the nature of the classification errors, as well as their quantities. Each cell in the confusion matrix represents the number of times that the gesture in the row is classified as the gesture in the column. It is shown that our HMVAN performs better than the method in [23].

**Table 10:** Confusion matrix for OPPORTUNITY dataset using our HMVAN.

		Predicted																	
		Null	Open Door 1	Open Door 2	Close Door 1	Close Door 2	Open Fridge	Close Fridge	Open Draw Washer	Close Draw Washer	Open Draw 1	Close Draw 1	Open Draw 2	Close Draw 2	Open Draw 3	Close Draw 3	Clean Table	Drink From Cup	Toggle Switch
Actual	Null	13832	6	5	5	3	24	15	5	2	10	13	5	4	22	39	7	58	9
	Open Door 1	10	76	0	10	0	0	0	0	0	0	0	0	0	0	0	0	0	0
	Open Door 2	7	0	155	0	2	0	0	0	0	0	0	0	0	0	0	0	0	0
	Close Door 1	8	15	0	78	0	0	0	0	0	0	0	0	0	0	0	0	0	0
	Close Door 2	10	0	0	0	130	0	0	0	0	0	0	0	0	0	0	0	0	0
	Open Fridge	111	0	0	0	0	253	22	2	0	0	0	0	0	0	0	0	0	1
	Close Fridge	41	0	0	0	0	19	210	0	1	0	0	0	0	0	0	0	0	0
	Open Draw Washer	61	0	0	0	0	6	0	99	4	1	0	0	0	0	0	0	0	0
	Close Draw Washer	43	0	0	0	0	2	0	10	79	0	0	0	1	0	0	0	0	0
	Open Draw 1	10	0	0	0	0	0	0	3	1	38	6	0	1	3	1	0	0	1
	Close Draw 1	20	0	0	0	0	1	0	0	0	8	46	0	0	0	0	0	0	0
	Open Draw 2	13	0	0	0	0	0	0	0	1	18	2	29	6	1	0	0	0	0
	Close Draw 2	5	0	0	0	0	0	0	0	2	1	5	4	25	0	3	0	0	0
	Open Draw 3	14	0	0	0	0	0	0	0	0	0	0	8	0	88	3	0	0	0
	Close Draw 3	6	0	0	0	0	0	0	0	0	0	0	2	9	5	80	0	0	0
	Clean Table	88	0	0	0	0	0	0	0	0	0	0	0	0	0	0	81	0	0
	Drink From Cup	143	0	0	0	0	0	0	1	1	0	0	0	0	0	0	0	397	0
	Toggle Switch	57	0	0	0	0	0	0	0	0	2	2	0	0	0	0	0	0	122

**Table 11:** Confusion matrix for OPPORTUNITY dataset using the method in [23].

		Predicted																	
		Null	Open Door 1	Open Door 2	Close Door 1	Close Door 2	Open Fridge	Close Fridge	Open Draw Washer	Close Draw Washer	Open Draw 1	Close Draw 1	Open Draw 2	Close Draw 2	Open Draw 3	Close Draw 3	Clean Table	Drink From Cup	Toggle Switch
Actual	Null	13752	5	8	6	5	39	18	14	29	2	0	1	1	40	20	2	114	8
	Open Door 1	17	51	0	28	0	0	0	0	0	0	0	0	0	0	0	0	0	0
	Open Door 2	15	0	111	0	38	0	0	0	0	0	0	0	0	0	0	0	0	0
	Close Door 1	10	22	0	69	0	0	0	0	0	0	0	0	0	0	0	0	0	0
	Close Door 2	9	0	7	0	124	0	0	0	0	0	0	0	0	0	0	0	0	0
	Open Fridge	130	0	0	0	0	220	34	4	1	0	0	0	0	0	0	0	0	0
	Close Fridge	49	0	0	0	0	76	146	0	0	0	0	0	0	0	0	0	0	0
	Open Draw Washer	108	0	0	0	0	4	0	45	14	0	0	0	0	0	0	0	0	0
	Close Draw Washer	75	0	0	0	0	4	0	30	26	0	0	0	0	0	0	0	0	0
	Open Draw 1	31	0	0	0	0	0	0	0	0	27	5	0	0	2	0	0	0	1
	Close Draw 1	40	0	0	0	0	0	0	0	0	19	16	0	0	0	0	0	0	0
	Open Draw 2	36	0	0	0	0	0	0	0	0	9	1	18	1	6	0	0	0	0
	Close Draw 2	14	0	0	0	0	0	0	0	0	3	1	13	5	9	0	0	0	0
	Open Draw 3	29	0	0	0	0	0	0	0	0	0	0	0	0	56	28	0	0	0
	Close Draw 3	9	0	0	0	0	0	0	0	0	0	0	0	0	51	42	0	0	0
	Clean Table	98	0	0	0	0	0	0	0	0	0	0	0	0	0	0	73	0	0
	Drink From Cup	194	0	0	0	0	0	0	0	0	0	0	0	0	0	0	0	349	0
	Toggle Switch	99	0	0	0	0	0	0	0	0	2	0	0	0	0	0	0	0	82

The auxiliary loss plays an important role in our network regularization. For strengthening the representation capability of each view, we calculate cross entropy losses to help regularize the network the learn the most discriminative features in each view of feature spaces. We also evaluate different weights on the main cross entropy loss and the auxiliary loss. The results of different weights seem almost same as the results of weight them equally. We infer that, since the type of main loss and the auxiliary loss are same and the task of them are both helping extract discriminative features, the performance of the model will not be influenced by different weights.

In terms of training time, there is not such a significant difference between our models and the other deep learning models, despite the complex attention mechanism included in the aggregation layer. Training HMVAN on the OPPORTUNITY dataset requires 342.5 minutes to converge while deepConvLSTM [11] requires 340.3 minutes. And the inference time of the whole dataset is about 7 seconds while deepConvLSTM [11] takes 6.68 seconds. The GPU used to train the model is NVIDIA GTX TITAN X. Recently, high-end mobile platforms already contain GPUs that can be used for general purpose processing [52]. A mobile processor, such as Qualcomm Snapdragon 855, comprises Adreno 640 GPU running at a maximum of 585 MHz and support OpenCL profiles for general purpose GPU computing. While cores differ in capabilities, the available computational power may well be sufficient for real-time recognition in upcoming mobile devices.

## 7 Conclusions and future works

In this paper, we propose a Hierarchical Multi-View Aggregation Network (HMVAN) for sensor-based human activity recognition (HAR). Compared with existing deep learning-based method, our method constructs multi-view feature spaces for each individual sensor from the point of white-box features and black-box features, and aggregate them into a unified representation by a hierarchical context. In addition, we propose three aggregation modules from the point of feature level, position level and modality level respectively. For feature level aggregation, we apply a non-local operation augmented with the *L*2-norm to explores the correlation between different features and aggregate them. Then, in the position level aggregation, we take the correlation of different sensor position into consideration by introducing the correlation feature, which can enhance the representation of each view. Finally, in the modality level aggregation, we conduct a soft attention mechanism to measure the discrimination of each view and fuse them for classification. Ablation study demonstrates the effectiveness of hierarchical multi-view architecture and the view aggregation modules. We extensively evaluated our method on 12 benchmark datasets, and our method is capable of achieving state-of-the-art performance on each dataset. At present, the sensor-based HAR method still relies heavily on labeled training samples. In future work, we plan to explore unsupervised or semi-supervised learning method for sensor-based HAR.
